# Who was the first to visualize the malaria parasite?

**DOI:** 10.1186/s13071-024-06145-4

**Published:** 2024-04-10

**Authors:** Emmanuel Drouin, Patrick Hautecoeur, Miles Markus

**Affiliations:** 1 Service de Neurologie, Groupe Hospitalier de l’Institut Catholique de Lille, GHICL, 115 Rue du Grand but, 59462 Lomme Cedex, France; 2 Wits Research Institute for Malaria, Faculty of Health Sciences, University of Witwatersrand, Johannesburg, South Africa; 3 School of Animal, Plant and Environmental Sciences, Faculty of Science, University of Witwatersrand, Johannesburg, South Africa

**Keywords:** History, Klencke, Lavéran, Malaria, *Plasmodium*

## Abstract

Human malaria, an ancient tropical disease, is caused by infection with protozoan parasites belonging to the genus *Plasmodium* and is transmitted by female mosquitoes of the genus *Anopheles*. Our understanding of human malaria parasites began officially in 1880 with their discovery in the blood of malaria patients by Charles Louis Alphonse Lavéran (1845–1922), a French army officer working in Algeria. A claim for priority was made by Philipp Friedrich Hermann Klencke (1813–1881) in 1843, who wrote a chapter entitled: “*Marvellous parallelism between the manifestations of vertigo and the presence of animalcule vacuoles in living blood*.” We should not lose sight of this old controversy, which is rarely mentioned in historical reviews on malaria.

**Graphical Abstract:**

**Figure:**
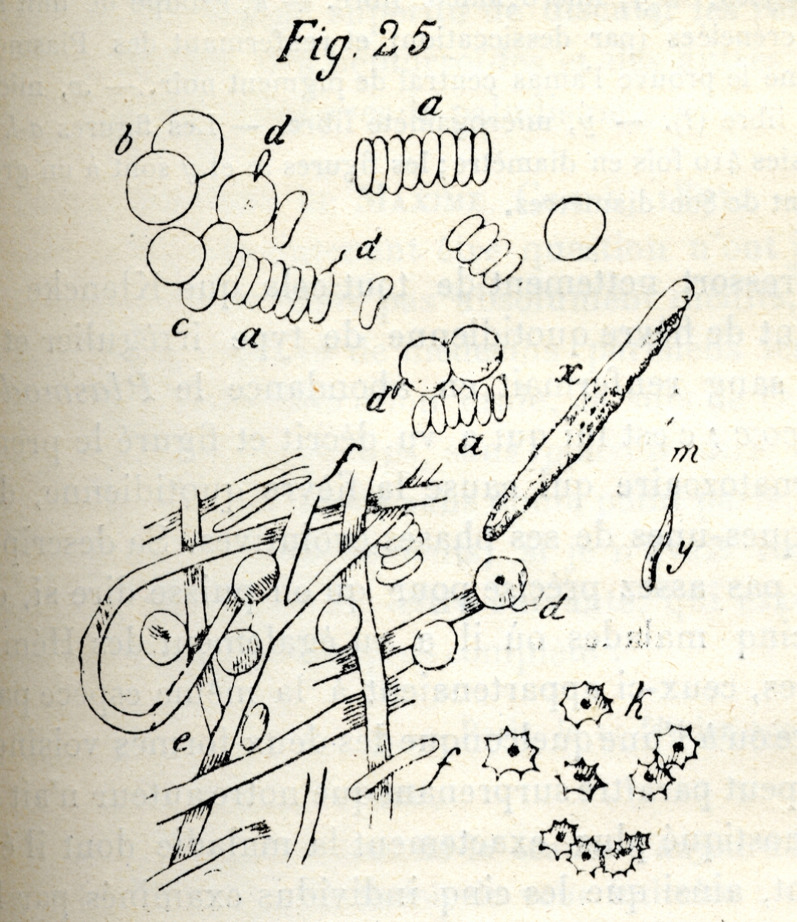

## Background

Human malaria is caused by infection with protozoan parasites belonging to the genus *Plasmodium* and is transmitted by female *Anopheles* spp. mosquitoes. The whole of the transmission cycle in culicine mosquitoes and birds infected with *Plasmodium relictum* was elucidated by Ronald Ross (1857–1932), a British army surgeon, in 1897. Malaria remains a leading cause of morbidity and mortality in humans worldwide. Our understanding of human malaria began officially in 1880, 144 years ago, with the discovery of the causative parasites in the blood of malaria patients by Alphonse Lavéran (1845–1922).

In 1882, he was able to convince Louis Pasteur (1822–1895) and Emile Roux (1853–1933), pre-eminent scientists of the time, of the protozoal etiology of malaria by demonstrating the parasite in a case of malignant malaria. In his important 1884 publication, “Traité des fièvres palustres,” Lavéran described his experience of a total of 480 cases.

## Discussion

On 23 November and then again on 28 December 1880, Alphonse Lavéran, a French military doctor and parasitologist, made the National Academy of Medicine in Paris aware of the existence of a specific parasite in the blood of some patients affected with fevers, thus demonstrating the parasitic etiology of malaria [1]. While serving as a surgeon in the French army in Algiers (Constantine), Lavéran discovered, on 6 November 1880, organisms in the blood of a soldier suffering from malaria. He noticed "moveable filaments or flagella, whose extremely rapid and varied movements left no doubt as to their nature." According to him, these elements had nothing in common with elements normally found in the blood, and he concluded that he was seeing organisms specific to malaria. Lavéran used fresh blood and dry objectives giving a maximum magnification of ×400 [2]. He found that the “flagella” detach easily and swim in the plasma. A full account of his studies was reported to the Académie des Sciences on 24 October 1881 [3] and was also presented before the Society of Medical Hospitals of Paris [4]. On 12 November 1881, the work was published in *The Lancet* [5]. E. Richard, a colleague of Lavéran stationed at Philippeville, Algeria, soon confirmed Lavéran's observations. The latter received the 1907 Nobel Prize in Physiology or Medicine. Lavéran named the infective organism *Oscilliaria malariae*; the genus name was subsequently changed to *Plasmodium*. Camillo Golgi (1843–1926) confirmed that malaria was caused by a protozoon and not a bacterium [6]. Excellent histories of malaria include [7].

At the time, there was protest against attribution of the discovery to Lavéran. Some detractors said that the intra-globular, so-called “spherical bodies" were not organized parasites but simply vacuoles, which are frequently encountered in the corpuscles on bad preparations. Scientists then wanted to invoke globular degeneration as an explanation for what was being seen. What becomes of the elements? How are they introduced into the host? How do they cause the disease? There were, as yet, no answers. It was natural that Lavéran's early observations on the hematozoon of malaria should be received with skepticism. Following controversies, Lavéran eventually had the satisfaction of seeing his discovery go unchallenged.

However, Lavéran was not in fact the first investigator to visualize the malaria parasite. It had already been observed before but without stimulating the interest the observations were deserved.

Already in the middle of the nineteenth century, many morbid anatomists including, among the first, Johann Friedrich Meckel (1821–1856) in 1847, had noted the presence of brown pigment in the organs of people who had died of pernicious fever. Meckel pointed out that the dark color of the spleen, liver, brain, or kidneys on autopsy of these cases was often associated with the accumulation of pigment in the blood. This was confirmed by Rudolf Virchow (1821–1902) in Germany. It is this pigment that formed the starting point for Alphonse Lavéran’s work. Meckel was probably observing malaria parasites without realising it because he did not mention malaria, thinking that the pigment was melanin.

**Fig. 1:**
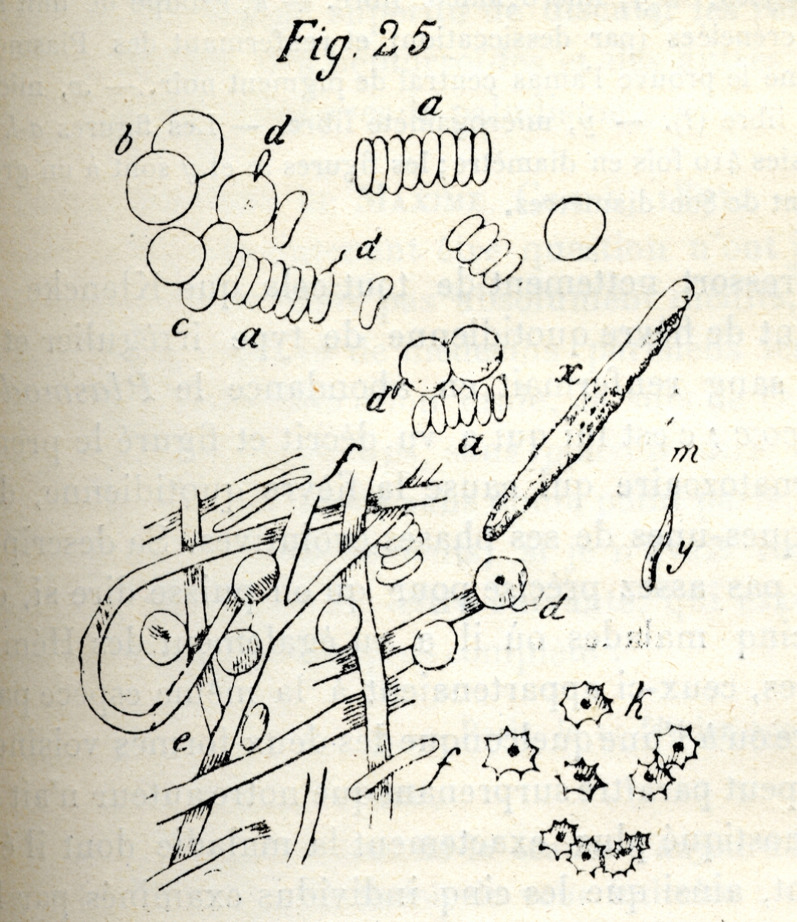
Klencke's Fig. 25 included in “Neue physiologische Abhandlungen auf selbständige Beobachtungen gegründet; für Aerzte und Naturforscher.” Leipzig; 1843. **a** Stacked red cells; **b** front view of red cells; **c** red blood cell stretched into the shape of a violin; **d** described by Klencke as small enterozoa adhering to red blood cells [8]; **e** fibrinous coagulum trapping red blood cells; **f** described by Klencke as larger, caterpillar-shaped, crawling enterozoa [8]; **k** group of eight crenate red blood cells; **x** described by Klencke as a caterpillar-shaped animal [8]. It is perhaps vaguely reminiscent of a free (extracellular) gametocyte of *Plasmodium falciparum*; **y** described by Klencke [8] as an enlargement of a smaller enterozoon, with the supposed head thereof at “**m**” in the representation. Drawings a–k at ×410 magnification, drawings x and y at ×800 magnification

A claim for priority was made by Philipp Friedrich Hermann Klencke (1813–1881) in 1843 [8]. Indeed, in his book he wrote a chapter entitled: “Marvellous parallelism between the manifestations of vertigo and the presence of animalcule vacuoles in living blood” (pp. 163–172), accompanied by his ambiguous Fig. 25 (Fig. 1). It is not clear that the structures illustrated by Klencke are Lavéran’s “flagella,” which are the microgametes of malaria parasites (see “Conclusions” below). Klencke portrayed red globules filled with black pigment and stated that such red globules are found in the blood of patients affected by vertigo (attacked by febrile fevers). As for the “growing body” and the “spherical body,” they were observed and illustrated as showing what might or might not have been the microgametes under development. Klencke clearly indicated that “animalcule vacuoles” arise on the surface of red blood cells.

Thus, affected by fever, Klencke would observe his own blood under a microscope and stated that “The entozoa usually surrounded a specific globule and did not detach themselves, so that I never saw the animalcules move from one globule to another. This is true of the smallest animalcules, while the largest were not connected to any globule.” The small animalcules “curved in rapid movement around the globule, attacking it at one point by an extremity corresponding to the head or detaching to reattach a little further away.” Klencke specified that every day when he was affected by the fever, he observed a large number of animalcules in his blood, 5 to 8 per 1000 globules, and they were extremely agile. “The parallelism of the appearance of entozoa and the attack of fever is truly astonishing,” he wrote. Klencke found that parallelism in five other patients who, like himself, were affected by fever.

## Conclusions

From a purely historical point of view, priority regarding the discovery may have been in Klencke's favor, but his description of what might have been exflagellation (formation of microgametes) is not clearly compatible with what he illustrated by drawings (Fig. 1). The morphology of microgametes in photomicrographs [9, 10] differs from that in the drawings in Fig. 1.

Klencke made the great parallelism between the presumed presence of organisms and the importance of vertigo, but without understanding its full significance as Lavéran had been able to do. It is very likely that Lavéran did not know about Klencke's observations, like the Germans themselves. Lavéran was probably not the first investigator to visualize the malaria parasite. We do not talk about Maxime Cornu (1843–1901) in 1871. Observations by Cornu have never been taken into consideration because they have remained unpublished. Camillo Golgi, a recipient of the Nobel prize for Medicine, was convinced of the value of Lavéran’s observations. Lavéran had the merit of understanding and indicating the general scope of his discovery [11].

## Data Availability

Not applicable.
